# Gentle Human Touch and Yakson: The Effect on Preterm's Behavioral Reactions

**DOI:** 10.5402/2012/750363

**Published:** 2012-06-25

**Authors:** Bahare Bahman Bijari, Sedigheh Iranmanesh, Fateme Eshghi, Mohammad Reza Baneshi

**Affiliations:** ^1^Faculty of Medicine, Kerman Medical University, Kerman 7616913555, Iran; ^2^Razi Faculty of Nursing and Midwifery, Kerman Medical University, Kerman 7616913555, Iran; ^3^Research Center for Modeling in Health, Kerman Medical University, Kerman 7616913555, Iran

## Abstract

*Objective*. Touch is one of the first strong positive senses that develop in neonate. Therapeutic touch could be considered as a complementary treatment in Neonate intensive care units (NICU). *Methods*. This quasi-experimental study was conducted to compare the effect of Yakson and GHT on behavioral reaction of preterm infants hospitalized in NICU in south-east of Iran. 90 preterm infants participated in this study. They are randomly divided into 3 groups: (1) Yakson group, *n* = 30, (2) GHT group, *n* = 30, (3) control group, *n* = 30. Each infant received the GHT and Yakson interventions twice a day for 5 days. Each session lasted 15 minutes. The control group received routine nursing care. *Results*. In interventional group, an increase was found in sleep state score after the Yakson and GHT intervention. Their awake and fussy states' scores decreased after both interventions. No significant difference was found between Yakson and GHT group in their behavioral state scores. *Conclusion*. The findings suggest that Yakson and GHT had soothing and calming effect on preterm infants and could be beneficial in nursing interventions.

## 1. Introduction

Preterm neonate—the classic high-risk neonate—is one born before completion of week 37 of gestation [1–3]. Medical and technological developments over the last few decades have increased the survival rates of infants born as preterm. Yet many of the procedures that are a necessary part of their postnatal care can be, by nature, painful and stressful [4, 5]. Since preterm infants have incomplete physical development, their admission in neonatal intensive care units (NICUs) provides them numerous stressors including painful stimuli, disruption of sleep, and excessive noise and light [5]. Sleeping and waking behaviors affect the development of preterm infants in various ways. According to [6], infants with neurological problems exhibit abnormal sleep patterns. Also sleep and wakefulness may have direct effects on brain development and learning that continue after the infant has been discharged home [6]. An important role of the neonatal nurse while providing care is to promote the preterm infant's growth and development through the control of the NICU environment [7]. Since preterm infants experience numerous stressors in the NICU environment, identifying interventions that have immediate soothing effect is essential [8].

Touch is crucial for optimal growth and development of preterm infants and one of the most developed senses provided by health care staff to relax preterm infants [5]. Therapeutic touch is considered as a complementary treatment [9]. Complementary therapies are attractive for nurses because they involve the whole human existence, and nurses are allowed to use them in their daily practice with no physician's order [10]. Touch as a complementary therapy provides nurses an opportunity to get close to the patients specifically when they give care to the neonates. Moreover therapeutic touch is a noninvasive treatment technique that requires no special equipment and technology. It can be easily combined with traditional medicine and thus decrease the cost of treatment, length of illness, and complications [11]. Thus, it is important for nurses to know the effects of touch on preterm infants in order to determine routine care and policies and to evaluate the potential of tactile stimulation as an effective intervention to promote overall growth and development [12].

One type of tactile stimulation which has a relaxing effect on preterm infants is gentle touch [8, 13]. In GHT, one hand is placed on the infant's head and the other hand is placed on the infant's abdomen to provide a relaxing effect to the preterm infant [5]. Similar to GHT, Koreans have traditionally believed that they could relieve their sick children's pain or discomfort by gently caressing their children on the aching body part. This caressing act is called “Yakson”, where “Yak” means medicine and “son” refers to hand [5]. The Yakson protocol for preterm infants includes the key aspects of traditional Yakson which are appropriate for preterm infants, such as warm hands, gentle touching without pressure, and slow hand movement. In Yakson, one hand is placed underneath the infant's back and the other hand is placed on the infant's abdomen and the abdomen is caressed [13].

 It seems that Yakson and GHT positively affects preterm's behavioral reactions. The work of [5] showed that 40 preterm infants receiving Yakson and GHT twice a day for 15 days had significantly greater reduction in 24-hour urinary cortisol and norepinephrine on day 16, compared to 20 other preterm infants receiving no touch therapy, and also after Yakson or GHT, the infants displayed an increased percentage of sleep states and a decreased percentage of awake and fussy states. In the other study, [13] compared the effect of Yakson and GHT on behavioral patterns. In both methods, the infants exhibited an increased percentage of sleep states and a decreased percentage of awake and fussy states. No significant difference was seen between two methods. Literature review showed that GHT and Yakson have benefits for preterm infant. In the Iranian context, no study was found to examine the effect of GHT/or Yackson on behavioral patterns. This study was conducted to compare the effect of these two methods on preterms' behavioral patterns in NICU in south-east of Iran.

## 2. Method

### 2.1. Design

The sample of neonates in this study was selected from one hospital under the supervision of Kerman University of Medical science. The study employed a quasi-experimental design and was conducted in Afzalipour hospital.

### 2.2. Sample

Ninety neonates (Yakson group, *n* = 30; GHT group, *n* = 30; control group, *n* = 30) who were admitted to neonatal intensive care unit participated in this study in Afzali pour hospital. The infants were randomly divided into 3 groups (control, Yakson, and GHT) following minimization approach. This allocation technique has been implemented considering baby's weight at birth and gestational age. Inclusion criteria were (1) a gestational age of 26–34 weeks at birth, (2) no congenital anomalies, (3) not having undergone surgery, (4) Apgar score more than 6 at 5 min, and (5) lack of medical condition contraindicating the running of Yakson or GHT such as symptomatic sepsis.

The GHT and Yakson interventions were provided for each infant twice a day for 5 days. Each session lasted 15 minutes. The time of intervention was relying on the meta-analysis of [14]. They stated that in previous studies, the time allocated to touch for preterm infants is between 15 and 30 minutes. The intervention was given to the infants twice a day. It seems that infants become more familiar with stimuli and can better remember these stimuli if they exposed to touch intervention twice a day [15].

A simple Yakson protocol was developed by Im and Kim (2009). The Yakson protocol for preterm infants includes some of the key aspects of the traditional Yakson (warm hands, touching without pressure, and slow hand movement) that are appropriate for preterm infants. Before the initiation of study one of the researchers (F. Eshghi) was trained for Yakson protocol and at the same time one of the NICU nurses was trained for GHT protocol. They were then evaluated by neonate subspecialist for correct administration intervention from the protocol. The protocol of Yakson used in this study consisted of the following procedures [5].

1. After wearing a clean inner gown, the researcher washed her hands and arms thoroughly with antimicrobial agents for 3 min.
2. The researcher warmed both of her hands using a radiant warmer until the temperature of the palms reached 93.2 8°F (34.0 8°C).
3. The researcher relaxed both arms and the muscles of both shoulders for 1 min and breathed deeply to concentrate Ki energy on their palms.
4. The researcher provided Yakson to preterm infants. Yakson lasted 15 min: hand resting (5 min), gentle caressing (5 min), and hand resting again (5 min). During the administration of Yakson, the palms and all fingers of the researcher constantly maintained close contact with the infants up to the limit that preterm infants did not feel pressure.
  - Hand Resting (5 min). While resting one hand on the chest and abdomen of a preterm infant while supporting the back of the preterm infant with the other hand, the researcher concentrated on her resting hands and envisioned that a healthy Ki was passing to the preterm infant. At that time, the researcher breathed slowly to maintain a relaxed state.
  - Gentle Caressing (5 min). In the same hand position, the researcher repeated caressing and resting for 5 min: caressing (1 min), resting (30 s), caressing (1 min), resting (30 s) and caressing (2 min). The researcher caressed the infant's chest and abdomen clockwise in a 1 cm diameter circular motion every 10 s.
  - Hand Resting (5 min). Researcher followed the same hand resting procedure as described previously.

The Gentle Human Touch (GHT) protocol was as follows: [8, 16].

1. After wearing a clean inner gown, the NICU nurses trained washed both hands and arms with antimicrobial agents for 3 min.
2. The NICU nurses trained warmed both of her hands using a radiant warmer until the temperature of the palms reached 93.2 8°F.
3. For 15 min, the NICU nurses trained placed the fingertips of one hand above the eyebrow line with the palm touching the preterm infant's crown while the other hand was rested on the lower abdomen of the infant encompassing the waist and the hip.

The person who was responsible for one intervention was not allowed to administer the other intervention. The interventions were provided for 5 consecutive days: 15 minutes in the morning and in the afternoon (9–11 a.m. and 3–5 p.m.). The other person who was NICU nurse was responsible for ABSS assessment. This person observed and assessed the infants' behavioral states 2 minute before initiation of interventions and 2 minute after that. She left the NICU ward during implementation of interventions. Data collection was done during 5 months (August 2011 to January 2012).

### 2.3. Instrument

The behavioral state of preterm infants was evaluated by the Anderson Behavioral State Scale (ABSS) [17]. This scale determines the behavioral state of an infant based on observations of respiratory regularity, opening or closing of the eyes, limb and trunk activity, and the intensity of crying. This scale measures 12 infant's behavioral states including 1; regular quiet sleep, 2; irregular quiet sleep, 3; active sleep, 4; very active sleep, 5; drowsy, 6; alert inactivity, 7; quiet awake, 8; active awake, 9; very active awake, 10; fussing, 11; crying and 12; hard crying. Scores from 1 to 5 indicate that the infant is sleeping. Scores 6–8 indicate that the infant is awake and calm and in the most suitable state for nursing activity. Scores from 9 to 12 indicate that the infant is in a state of restless activity or fussiness, which take substantial energy. We analyzed the ABSS score as a continuous variable.

Since physiological stabilization and developing sequelae for infants lasts 7 days [12], Yakson and GHT interventions began 7 days after birth. Additionally, whenever one of the preterm infants was touched as part of any nursing care (feeding or diaper changing), the GHT or Yakson treatments were postponed by 1 h. According to [5] in case that infant indicated any sign of distress (decreased heart rate or oxygen saturation levels) during a GHT or Yakson intervention, the intervention was ended and nursing care was provided. Once the preterm infant became stable, the intervention was delivered.

For translation of ABSS scale from English into Farsi, the standard forward-backward procedure was applied. Translation of the items was independently performed by two professional translators and then temporary versions were provided. Afterward they were back translated into English, and, after a careful cultural adaptation, the final versions were provided.

### 2.4. Ethical Consideration

There was an approval from the heads of NICU prior to the collection of data. The study proposal also was reviewed and approved by center's office of Research Ethics in Kerman Medical University (ethic code: K/90/342). The written informed consent forms were signed by parents. The consent form explained that participation was completely voluntary, and they can withdraw from the study at any time. They were informed about the purpose of study and procedure, both verbally and with written information. To secure confidentiality there was no personal information on the scale.

### 2.5. Data Analysis

Using SPSS software version 18.00 data were analyzed. Descriptive statistics was used to determine mean, SD, frequency, and percent of categorical variables. Chi-square and ANOVA tests were applied to compare distribution of demographic characteristics across two intervention and control groups. The main outcome of study was ABSS score. There were multiple measurements per subject (12 measures per subject); therefore there is a degree of similarity or correlation between measurements of the same subject. Impact of variables on ABSS score was evaluated applying Mixed Model. This technique takes into account dependency of data and assesses trend in ABSS score over time and influence of independent variables on it. A *P* value less than 0.05 was considered as statistical significance level.

## 3. Results

### 3.1. Sample Characters

A descriptive analysis of the background information (Table 1) indicated that the premature infants belonged to the gestational age 26–34 weeks with a mean age of 31 weeks. They were mostly female (56.7%). About 67% of participants were born with C/S (cesarean section) method. Three groups' mean of birth weight was almost 1650 g. The mean score of Apgar for three groups was 8. *χ* ^2^ analyses and ANOVA showed that there were no significant differences among 3 groups in demographic variables (Table 1).

### 3.2. Behavioral State

**Figure 1:**
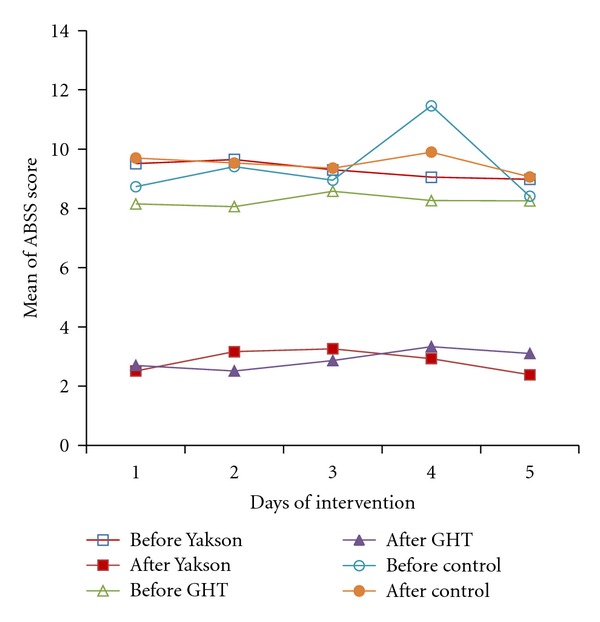
Comparison mean of ABSS score measured before and after intervention in Yakson, GHT, and control groups in 5 days of intervention.

The results indicated no significant correlation between type of delivery/sex and ABSS scores. The mean (SE and *P* value) of ABSS score in the infant with S/C type of delivery was 6.74 (0.11, 0.7). For infants with nvd type of delivery, it was 6.8 (0.14, 0.7). The mean (SE and *P* value) of ABSS score for male infant was 6.87 (0.13, 0.15), and for female infant it was 6.67 (0.11, 0.15). The results of the study also demonstrated that birth weight (*P*  value = 0.35) and Apgar score (*P*  value = 0.15) of infants have no significant effect on their ABSS scores. In the study, the infants' behavioral reactions were observed before and after the period of touch. Mixed Model statistical test showed that infants' ABSS scores are different between three groups. There was a correlation between the groups of infants and the change of ABSS scores. The Mean (SE) of ABSS score for Yakson group, GHT group, and control group was 4.83 (0.14), 4.85 (0.14), and 10.63 (0.14). As indicated in Table 2, a significant difference was found between the effect of interventional groups and control group on infants' sleep states (*P*  value < 0.001). It means that infants' sleep state increased after the interventions (Table 3). No significant difference was found between Yakson and GHT groups in behavioral states' scores (*P*  value = 1.00) (Table 2). An increase was found in the infants' sleep state after Yakson and GHT interventions (Figure 1). A decrease was also found in the infants' awake and fussy states after Yakson and GHT interventions (Figure 1). As it is shown in Figure 1 and Table 3, infants in control group experienced more fussy states compared to both interventional groups. In the analysis, the changes of ABSS scores were checked in the consecutive days of interventions. The mean (SE) of ABSS score in the first and second days of intervention was 8.97 (0.36) and 9.05 (0.33). In the third day of intervention mean (SE) of ABSS had a significant reduction 5.58 (0.18). In the forth and fifth days of intervention, no significant change was seen in mean (SE) of ABSS scores. The result of analytical analysis indicated no significant different in infants' behavioral states between first and second days of intervention (*P*  value = 1.00). A significant difference was found between behavioral states' scores of first and second days with third, fourth, and fifth day of interventions (*P*  value < 0.001). Infants' sleep states' score increased in third, fourth, and fifth days of interventions. However no significant difference was found in infants' behavioral states in days 3, 4, and 5 of interventions.

## 4. Discussion

The aim of this study was comparison of effect of Yakson and GHT on behavioral states among preterm infants. According to the results, both Yakson and GHT groups compared with control group indicated more sleep states after interventions compared to that before the intervention (Figure 1). Similarly, previous researchers [5, 13] found that these interventions positively affect infant s' sleep states. The work of [5] also reported that these interventions decreased the secretion of stress hormones in preterm infants. In control group, the fussy state's score of infants was more than that in both interventional groups. This result can be supported by early studies that showed infants in interventional group experienced a better behavioral state compared to those in control group [5, 13]. It is well known that most of touch that preterm infants receive in the NICU is related with medical or nursing procedures. The infants in control group received no intervention but they routinely received procedural touch as a nursing care. So this type of touch as a routine manipulation could negatively affect on infants' behavioral state.

The findings also indicated no difference between effect of Yakson and GHT on infants' sleep states after administration of these interventions (Table 2 and Figure 1). This finding is inconsistent with result of previous study [13]. They found that calming effect of Yakson intervention on infants is significantly greater than that of GHT intervention. This difference could be related to the different sampling or sample size they used in their study. According to the results, after GHT intervention sleep states were increased. This finding is consistent with previous researches [5, 8, 12, 13, 18–20] where they found more sleep states during and after GHT compared to those before GHT. This finding therefore supports previous studies that reported positive effect of GHT on preterm infants' sleep state.

According to the results, after GHT and Yakson interventions infants' awake and fussy states decreased (Table 3). This finding echoes the results of earlier studies that showed positive effect of GHT and Yakson on awake states after interventions [5, 8, 13] and on awake as well as fussy states after interventions [5, 13]. This finding indicated that GHT and Yakson interventions promote infants' comfort, diminish their stress, and help them to be calm during their hospitalization. According to the results, a significant difference was found between infants' behavioral states in days 1 and 2 and days 3, 4, and 5 of intervention. The infants' sleep states increased in days 3, 4, and 5 of intervention compared to days 1 and 2. According to the results, no significant difference was found between infants' sleep state in days 3, 4, and 5. In this study the interventions were done in five days. In previous related studies, the interventions were administered for more than 5 days. For example, in [5] study, the intervention continued for 15 days. In these studies, there is no report about the day of study, for example, its relation with the intervention or its effect on infants' sleep state. Therefore, the difference found in this study between days of intervention on infants' behavioral state cannot be supported by and does not support previous studies. This finding can be explained by the fact that in days 1 and 2 the infants are not well familiar with these interventions. In days 3, 4 and 5 of interventions, the infants become familiar with the interventions and interventions sufficiently affects infants' behavioral states.

## 5. Conclusion

The results of this study showed that Yakson and GHT increase infants' sleep states and can reduce stress and energy expenditure and consequently decrease O_2_ dependency during the early weeks in the preterm infants hospitalized in the NICU. They consequently can cause growth and development in the preterm infants. Of course, further research needs to be done in order to examine the effect of Yakson and GHT on later developmental outcomes in Childhood period. Since touch is one of the first strong positive senses in neonate, possibly Yakson and GHT enhance sensory maturation and thereby promote more optimal behavioral organization at the time of hospital discharge. The study suggests that the appropriate effects of Yakson and GHT should be assessed in the different situations, for example painful procedures such as venipuncture, intubation, and suctioning. According to the findings of this study, Yakson and GHT could be one of the safe interventions that nurses use for preterm infants in order to enhance their development. Nurses working in NICU and parents need to be educated on how to provide Yakson or GHT for preterm infants. All information about the risks and benefits as well as the best interventional strategies before this intervention should be included in this educational program. Further study suggests to examine NICU nurses' views about two types of touch therapy (GHT and Yakson), their benefits, and their disadvantages as well. It also suggests a study to compare the effect of these two types of touch on preterm infants when they were administered by NICU nurses and parents.

## Figures and Tables

**Table 1:** Homogeneity test for general characteristics among the groups (*N* = 90).

Characteristics	Yakson (*n* = 30)	GHT (*n* = 30)	Control (*n* = 30)	*P*
	*N* (%) or *M* (SD)	*N* (%) or *M* (SD)	*N* (%) or *M* (SD)	
Sex				
Male	13 (43.3)	13 (43.3)	13 (43.3)	1.0
Female	17 (56.7)	17 (56.7)	17 (56.7)	
Delivery type				
Nvd	9 (30)	8 (26.6)	13 (43.3)	0.35
S/C	21 (70)	22 (73.4)	17 (56.7)	
Gestational age (week)	31.70 (2.16)	31.70 (1.74)	31.33 (1.95)	0.70
Birth weight (gram)	1569.66 (315.98)	1722.66 (450.24)	1631.66 (407.36)	0.32
Apgar score (5 min)	8.7 (.98)	8.3 (1.2)	8.7 (1.11)	0.22

**Table 2:** Behavioral states score of three groups in the preterm infants (Yakson = 30, GHT = 30 and control = 30).

	Groups	Mean	Std. Error	Groups		Sig.
Behavioral state	Yakson	4.834	.140	Yakson	GHT	1.000
					Control	.000
	GHT	4.852	.144	GHT	Yakson	1.000
					Control	.000
	Control	10.630	.140	Control	Yakson	.000
					GHT	.000

**Table 3:** Behavioral state scores for preterm infants before and after the intervention of 5 days (*N* = 90; Yakson *n* = 30; GHT *n* = 30; control *n* = 30).

	Group	1day	2day	3day	4day	5day
		Mean(SD)	Mean (SD)	Mean (SD)	Mean (SD)	Mean (SD)
Behavioral state	Yakson					
	Before	9.51 (2.09)	9.65 (1.54)	9.30 (2.31)	9.05 (2.41)	8.98 (2.10)
	After	2.51 (1.41)	3.16 (1.37)	3.26 (1.62)	2.93 (1.41)	2.38 (1.08)
	GHT					
	Before	8.15 (2.62)	8.06 (3.26)	8.58 (2.77)	8.26 (2.60)	8.25 (2.51)
	After	2.70 (1.95)	2.51 (1.64)	2.86 (1.82)	3.33 (1.78)	3.10 (1.55)
	control					
	Before	8.73 (1.98)	9.41 (1.58)	8.95 (2.07)	11.46 (9.21)	8.41 (2.46)
	After	9.70 (1.05)	9.53 (1.31)	9.36 (1.37)	9.90 (.73)	9.06 (1.38)
